# IL13Rα1 prevents a castration resistant phenotype of prostate cancer by targeting hexokinase 2 for ubiquitin-mediated degradation

**DOI:** 10.20892/j.issn.2095-3941.2020.0583

**Published:** 2021-10-18

**Authors:** Tingting Feng, Jing Wang, Kai Cheng, Qiqi Lu, Ru Zhao, Shiguan Wang, Qingyun Zhang, Luna Ge, Jihong Pan, Guanhua Song, Lin Wang

**Affiliations:** 1 Department of Pathology, School of Basic Medical Sciences, Shandong University, Jinan 250012, China; 2 Department of Pathology, The Fourth People’s Hospital of Jinan, Jinan 250031, China; 3 Department of PET-CT, Shandong Cancer Hospital and Institute, Shandong First Medical University and Shandong Academy of Medical Sciences, Jinan 250002, China; 4 The Second Hospital, Cheeloo College of Medicine, Shandong University Medical School, Jinan 250012, China; 5 Biomedical Sciences College & Shandong Medicinal Biotechnology Centre, Key Lab for Biotech-Drugs of National Health Commission, Key Lab for Rare & Uncommon Diseases of Shandong Province, Shandong First Medical University & Shandong Academy of Medical Sciences, Jinan 250002, China; 6 Department of Biochemistry and Molecular Biology, Shandong University School of Basic Medical Sciences, Jinan 250012, China; 7 Institute of Basic Medicine, Shandong Academy of Medical Sciences, Shandong First Medical University & Shandong Academy of Medical Sciences, Jinan 250002, China; 8 Department of Oncology, The First Affiliated Hospital of Shandong First Medical University, Jinan 250014, China

**Keywords:** Prostate cancer, castration resistance, IL13Rα1, apoptosis, glycolysis

## Abstract

**Objective:**

Androgen deprivation therapy (ADT) is still the principal treatment option for prostate cancer (PCa). In addition to reactivation of androgen receptor signaling, the resistance of PCa to apoptosis during ADT also contributes to castration resistant PCa (CRPC). A previous study reported that gene transfer of IL-13Rα2 into PCa cells sensitized the cells to the IL-13R-targeted cytotoxin IL13Rα1, leading to apoptosis. Compared with IL-13Rα2, IL13Rα1 is more constitutively expressed in PCa cells, but its function in PCa remains to be established.

**Methods:**

We determined the role and expression of IL13Rα1 in PCa cancer cells using western blotting, flow cytometry, and cell proliferation assays. Co-immunoprecipitation and mass spectrometry were used to identify the proteins that interacted with IL13Rα1, to elucidate its function.

**Results:**

In this study, we showed that IL13Rα1 was selectively suppressed in androgen-deprived PCa cells and that its suppression tended to be associated with poor prognoses of PCa patients. IL13Rα1 overexpression promoted apoptosis and inhibited tumor growth under androgen-deprived or castrated conditions (*P* < 0.01). Mechanistically, IL13Rα1 recruited and facilitated ubiquitin protein ligase E3C-mediated ubiquitination and degradation of hexokinase 2 (HK2), resulting in glycolytic inhibition and eventually leading to PCa cell apoptosis. Furthermore, our data revealed that mutated ataxia-telangiectasia kinase phosphorylated and facilitated the selective ubiquitin proteasome-mediated degradation of HK2. Notably, IL13Rα1-overexpressing PCa cells were more susceptible to apoptosis and exhibited reduced tumor growth after exposure to the HK2 inhibitor, 2-deoxy-D-glucose (*P* < 0.01).

**Conclusions:**

Our data identified a tumor suppressor role for IL13Rα1 in preventing the resistance of PCa cells to apoptosis during androgen deprivation by inhibiting glycolysis. IL13Rα1-mediated signaling involving HK2 may therefore provide a novel treatment target and strategy for CRPC.

## Introduction

Although prostate cancer (PCa) is initially sensitive to hormonal manipulation, resistance to androgen deprivation therapy (ADT) ultimately occurs^1^. Resistance to apoptosis is one of the mechanisms by which cancer cells overcome the toxicity of therapeutic agents^2^. Apoptosis is the best understood type of regulated cell death, and has long been considered irreversible. Multiple studies have shown that the inability of PCa to undergo apoptosis leads to transition to castration resistant PCa (CRPC)^3^. Overcoming the apoptotic resistance induced by ADT may therefore serve as an important strategy for PCa treatment.

IL-13Rα1 is an ubiquitously expressed low affinity interleukin (IL)-13 receptor (IL-13R), but after binding to IL-13, it recruits IL-4Rα and forms a high affinity IL-13R complex (type II IL-13R) and activates the Janus-activated kinase (JAK)-STAT pathway^4^. The interaction of IL-13 with IL13Rα 1 increases the susceptibility of human and murine cells to oxidative stress^5^. To target IL-13R, a recombinant fusion IL-13R cytotoxin was developed^6^. It was reported that a monoclonal antibody against IL13 prevented its binding to IL13Rα1 and IL13Rα 2 and inhibited esophagitis progression^7^. Notably, gene transfer of IL-13Rα2 induced more apoptosis when PCa cells were simultaneously exposed to a IL-13R-targeted cytotoxin. A previous study reported that IL13Rα1 was more constitutively expressed in PCa cells than IL-13Rα2 or interleukin 4 receptor (IL4Rα)^8,9^ although its exact role remains to be elucidated. IL-13Rα1 has no intrinsic kinase activity, but is constitutively associated with tyrosine kinase 2 (Tyk2) *via* its proline-rich Box-1 region in the cytoplasm^10^. Furthermore, IL13Rα1 is required for IL13 to mediate the pathological features associated with asthma and lung injury, which involves fibroblasts, eosinophils, and dendritic cells^11^. Importantly, the increased pathology in bleomycin-treated IL13Rα1^-/-^ mice may be largely independent of IL13^12^. Because the cytoplasmic domain of human IL13Rα1 contains 2 tyrosine residues, Y402 and Y405^10^, which might serve as docking sites for additional signaling intermediates, it is important to elucidate its downstream signaling pathways.

The vast majority of solid cancers undergo the Warburg effect, a phenomenon of increased glucose uptake and glycolysis despite aerobic conditions^13^. By catalyzing the phosphorylation of glucose, hexokinase 2 (HK2) facilitates glucose entry into cells, initiating glycolysis and glucose-linked pathways^14^. In addition, mitochondrial-associated HKs antagonize the binding of apoptotic B-cell lymphoma 2 family members at the mitochondrial outer membrane and prevent mitochondrial apoptosis^15^. In PCa, elevated levels of mitochondrial HK2 and its enzymatic activity have been observed in androgen-deprived tumors^16^. Combination treatment with the glucose analogue 2-deoxy-D-glucose (2-DG), which inhibits HK2 activity, could resensitize PCa cells to enzalutamide, suggesting that it would be helpful to provide an additional therapeutic approach by characterizing the role of HK2 in PCa. In this study, we designed a series of functional and molecular assays to characterize the role of IL13Rα1 in PCa, and found that IL13Rα1-mediated glycolytic inhibition was a potential treatment for PCa, especially CRPC.

## Materials and methods

### Cell culturing and treatments

LNCaP and C4-2B were obtained from Dr. Jindan Yu (Northwestern University, Chicago, IL, USA) in 2015. VCaP and HEK293T were purchased from the American Type Culture Collection (Manassas, VA, USA) and authenticated again by short tandem repeat analysis before and after our study. The cumulative culture length of cells between thawing and use in this study was less than 15 passages. All newly revived cells were found to be free of mycoplasma contamination.

### Reagents, plasmids, and lentivirus transduction

MG132 and cycloheximide (CHX) were purchased from Calbiochem (San Diego, CA, USA). Leupeptin, CoCl_2_, thapsigargin, and tunicamycin were purchased from Sigma-Aldrich (St. Louis, MO, USA). ATM inhibitor and STAT6 inhibitor were purchased from Selleck (Houston, TX, USA). IL-13 was purchased from R&D Systems (Minneapolis, MN, USA). Anti-human IL13-IgG was purchased from InvivoGen (San Diego, CA, USA). The 2-DG was from Seahorse Bioscience (Billerca, MA, USA). Myc-tagged-IL13Rα1 wild type (wt), Myc-tagged-IL13Rα1 Box1-delected IL13Rα1, hemagglutinin (HA)-tagged-HK2, FLAG-tagged-HK2, HA-tagged-UBE3C, HA-tagged-Tyk2-wt, FLAG-tagged-wt ATM, and FLAG-tagged-active ATM-wt (a) were constructed and cloned into pcDNA3.1/Zeo (+) (Thermo Fisher Scientific, Waltham, MA, USA) by Cyagen Biosiences (Guangzhou, China). The expressions of the above constructs are shown in **Supplementary Figure S1**. Enzymatically inactive mutant (mt) FLAG-tagged-ATM, FLAG-tagged-mt HK2 (S778A), HA-tagged-mt Tyk2 (K930R), Myc-tagged-mt IL13Rα1 (Y402A), and Myc-tagged-mt IL13Rα1 (Y405A) were generated using the QuikChange site-directed mutagenesis kit (Stratagene, San Diego, CA, USA) as previously described. ATM (31985) and TyK2 (23908) were purchase from Addgene (Watertown, MA, USA). HA-tagged-ubiquitin was kindly provided by Chengjiang Gao (Shandong University, Shandong, China). Lentivirus products were constructed in 293T cells by co-transfection with psPAX2, pMD2.G, and lentiviral construct (pLentiCMV/TO Puro DEST) expressing wt IL13Rα1 (Lv-IL13Rα1), wt IL13Rα2 (Lv-IL13Rα2), mt IL13Rα1 (Y402A), mt IL13Rα1 (Y405A), and wt Tyk2 (Lv-Tyk2) using Lipofectamine 2000 (Invitrogen, Carlsbad, CA, USA).

### Cell culture, siRNA transfection, cell viability, and apoptosis

The siRNAs were purchased from Ruibo Biosciences (Guangzhou, China). For transient RNA interference, the cells were transfected with siRNA using Hiperfect Transfection Reagent (Qiagen, Hilden, Germany) according to the manufacturer’s instructions. The silencing efficiency siRNAs targeting IL13Rα1, IL4Rα, L13Rα2, ATM, ubiquitin protein ligase E3C (UBE3C), or Tyk2 were measured using western blotting (**Supplementary Figure S1**) and the 2 showing silencing efficiencies were selected and combined at equal concentrations for the following experiments. The sequences are shown in **Supplementary Table S1**. PCa cells were transfected with siRNA (100 nM), Lv-IL13Rα1, or their controls with or without hormone-starved media containing 10% charcoal stripped fetal bovine serum. Next, cell viability and apoptosis were detected using MTS cell proliferation assay and Annexin V-FITC apoptosis kits.

### The 2-NBDG [2-N-(7-nitrobenz-2-oxa-1,3-diazol-4-yl)(amino)-2-deoxyglucose] glucose uptake and lactate production

Cells were plated in 24-well plates (2 × 10^5^ cells/well) and 2-NBDG was added at 10 µM final concentration and incubated for 1 h at 37 °C. The fluorescence intensity was immediately measured in a microplate reader (Spectramax ID3; Molecular Devices, San Jose, CA, USA) at an excitation wavelength of 485 nm and an emission wavelength of 530 nm. After being internalized by cells, 2-NBDG is converted to a non-fluorescent derivative (2-NBDG metabolite). An estimate of the overall glucose uptake was obtained by quantifying the fluorescence. Lactate levels were measured using a Lactate Assay Kit (Biovision, Milpitas, CA, USA) according to the manufacturer’s instructions.

### Extracellular acidification and oxygen consumption rate assays

The extracellular acidification rate (ECAR) and cellular oxygen consumption rate (OCR) were determined using the Seahorse XFe 24 Extracellular Flux Analyzer (Seahorse Bioscience). Experiments were performed according to the manufacturer’s instructions. After the indicated treatments, ECAR and OCR were determined using the Seahorse XF Glycolysis Stress Test Kit and Seahorse XF Cell Mito Stress Test Kit, (Seahorse Bioscience), respectively. Data were analyzed using Seahorse XF software (Seahorse Bioscience). The OCR is expressed in pmole/minute, and the ECAR is expressed in mpH/minute.

### The glutathione S transferase (GST) pull-down assay and mass spectrometry (MS)

Bacterially-expressed GST-IL13Rα1 or control GST (both, 500 µg) bound to Glutathione-Sepharose 4B beads (Amersham Pharmacia, Piscataway, NJ, USA) was incubated with equal amounts of whole cell lysates of PCa cells at 4 °C overnight. The washed complexes were eluted by boiling in SDS sample buffer, separated by SDS-PAGE, and then incubated with anti-GST-conjugated beads for 24 h in a cold room. The immunoprecipitates were identified by MS (BGI, Hongan, China).

### Western blotting and immunoprecipitation (IP)

The cells were collected and lysed with RIPA buffer plus protease inhibitor cocktail (Roche Applied Science, Penzberg, Germany)^17^. For the chase assay of protein stability, CHX (100 µg/mL), leupeptin (100 µM), and/or MG132 (20 µM) were used for the indicated times. For immunoprecipitation (IP), cleared lysates were incubated with the indicated antibodies (1 µg) and subsequent Protein A-coupled magnetic Dynabeads (Thermo Fisher Scientific). The samples were subjected to SDS-PAGE, and the resulting bands were transferred onto polyvinylidene difluoride membranes for visualization^18^. Primary antibodies used in our study are described in **Supplementary Table S2**. The secondary antibodies used were horseradish peroxidase-coupled anti-mouse IgG and anti-rabbit IgG (GE Healthcare, Chicago, IL, USA). Antibody binding was detected by enhanced chemiluminescence with hyperfilm ECL or an RGB 600 Imager (GE Healthcare).

### Immunofluorescence (IF)

For immunofluorescence, the cells were sequentially probed with primary antibodies and fluorescently-labeled secondary antibodies (Jackson Immunoresearch, West Grove, PA, USA). Images were captured using a confocal microscope (FV3000; Olympus, Tokyo, Japan).

### Ubiquitination assay

For the ubiquitination assay of PCa cells, the cells were transfected with Lv-IL13Rα1 and siUBE3C, or their controls. For the ubiquitination assay, HEK 293T cells were transfected with plasmids as indicated using Lipotamine 2000 (Invitrogen). The lysates were then sonicated for 15 s on ice and boiled at 100 °C for 10 min. The boiled samples were diluted with lysis buffer containing 0.1% SDS and then centrifuged at 12,000 rpm for 5 min. Denatured lysates were immunoprecipitated with anti-FLAG antibody and analyzed using immunoblots with anti-k48 or anti-k63 linked polyubiquitin.

### Animal treatment

Male athymic nude mice (nu/nu; 4-weeks-old) were purchased from Weitonglihua Biotechnology (Beijing, China) and maintained in a specific pathogen-free environment. The animal studies were approved by the Institutional Animal Care and Use Committee of the Biomedical Sciences College & Shandong Medicinal Biotechnology Centre (SMBC17LL007), and the guidelines were strictly followed. For both PCa progression and the CRPC model, male athymic nude mice were injected subcutaneously with 5 × 10^6^ stably IL13Rα1-overexpressing C42B [suspended in Matrigel (BD Biosceinces, San Jose, CA, USA)]. For treatment, mice were randomly selected using 2-DG or vehicle daily for 5 days a week for 8 weeks. Each experimental group consisted of 10 mice. The tumor response measurements were conducted as previously described^18^. Animals were sacrificed after 7 weeks of treatment and all mice survived to the end of treatment. Data points were expressed as the mean tumor volume ± SD.

### Statistical analysis

Statistical significance was assessed using an unpaired two-tailed Student’s *t*-test. Correlation significance was assessed using Pearson’s correlation coefficient test. *P*-values < 0.05 were considered statistically significant. Data are presented as the mean ± S.D. Statistical analysis was performed using Prism 5.0 software (GraphPad Software, La Jolla, CA, USA).

## Results

### IL13Rα1 is required to promote apoptosis of PCa cells

**Figure 1:**
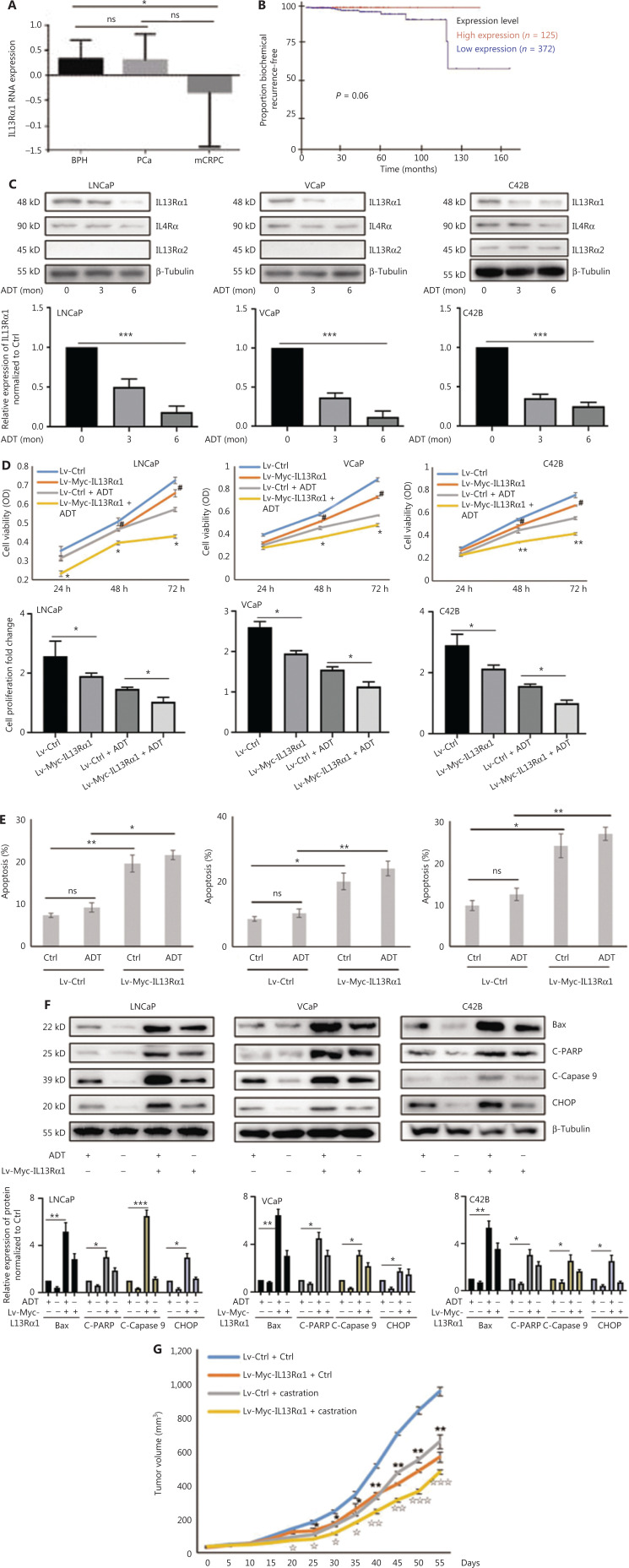
IL13Rα1 is responsive to androgen deprivation and promotes apoptosis of prostate cancer (PCa) cells. (A) The expression of IL13Rα1 in different prostate samples from the GSE6956 dataset. *P < 0.05; ns, not significant (two-tail Student’s *t*-test). (B) The correlation between IL13Rα1 expression and biochemical recurrence-free survival (log-rank test) in The Cancer Genome Atlas cohort from 497 PCa cases. (C) Analyses of IL13Rα1, IL-4Rα, and IL13Rα2 in LNCaP, VCaP, and C42B cells with androgen deprived treatment (ADT) for the indicated month. The bar graphs are presented as a fold ratio to the control. ***P < 0.001. (D) Cell viability was measured by the MTS assay in LNCaP, VCaP, and C42B cells with the introduction of the lentiviral-Myc-tagged IL13Rα1 (Lv-Myc-IL13Rα1) or its control (Lv-Ctrl). *P < 0.05; **P < 0.01: Lv-Myc-IL13Rα1 *vs.* Lv-Ctrl; ^#^*P* < 0.05: Lv-Myc-IL13Rα1+ADT *vs.* Lv-Ctrl+ADT (two-tail Student’s *t*-test). Graphs show fold changes compared with day 0. (E) The apoptosis of LNCaP, VCaP, and C42B cells containing Lv-Myc-IL13Rα1 or Lv-Ctrl was assessed by Annexin V staining with or without ADT for 3 days. *P < 0.05; **P < 0.01; ns, not significant (two-tail Student’s *t*-test). (F) Whole cells lysates were also subjected to western blotting using anti-Bax, anti-cleaved PARP, anti-cleaved caspase 9, or anti-CHOP. The band intensities underneath gel images were measured using ImageJ software (NIH, Bethesda, MD, USA). The graphs of the gel images were derived using ImageJ software. β-Tubulin was used as the loading control. *P < 0.05; **P < 0.01; ***P < 0.001. (G) Male Balb/c athymic nude mice with or without castration were injected subcutaneously with a total of 5 ×106 cells/100 mL of C42B transduced with Lv-Myc-IL13Rα1 or Lv-Ctrl. Tumor volumes were measured on the indicated days. The number of animals in each group was 10. *P < 0.05, **P < 0.01: Lv-Myc-IL13Rα1 *vs.* Lv-Ctrl; ^I^*P* < 0.05, ^II^*P* < 0.01, ^III^*P* < 0.001: Lv-Myc-IL13Rα1+Castration *vs.* Lv-Ctrl+Castration (two-tail Student’s *t*-test). Data are from 2 or 3 independent experiments (C, F, and G) or are representative of 3 independent experiments with similar results (D and E; mean ± SD).

To characterize the clinicopathological characteristics of IL13Rα1 expression in PCa patients, we first performed *in silico* analysis of IL13Rα1 mRNA expression using published datasets. **Figure 1A** shows that the IL13Rα1 mRNA level was significantly lower in CRPC tissues than in primary PCa tissues. Notably, an association between IL13Rα1 overexpression and biochemical recurrence was also observed in the GSE70769 dataset (**Figure 1B**; *N* = 497, *P* = 0.06), although this association did not reach statistical significance (**Figure 1B**). To determine the potential pathophysiological function of IL13Rα1 in CRPC, we determined the effects of ADT on IL13Rα1 expression. IL13Rα1 protein levels decreased more (**Figure 1C**) than IL4Rα and IL13Rα2 levels under androgen deprivation conditions. We then determined the effects of IL13Rα1 on cell proliferation and apoptosis. The results showed that overexpressing IL13Rα1 by lentivirus transfection resulted in significant inhibition of cell proliferation (*P* < 0.05) (**Figure 1D**) but induction of apoptosis (**Figure 1E**), which was greater (*P* < 0.01) in the presence of ADT. In addition, the expressions of apoptotic markers were easily induced by ADT after the introduction of exogenous IL13Rα1 into LNCaP, VCaP, and C42B cells (**Figure 1F**). We also determined the *in vivo* role of IL13Rα1 in PCa. Compared to a vector control (VCtrl), ectopic expression of IL13Rα1 significantly inhibited (*P* < 0.01) the growth of C42B tumor xenografts under both normal and castrated conditions (**Figure 1G**). In addition, the introduction of IL-13Rα2 or silencing of IL-4Rα in PCa cells did not affect the increased apoptosis induced by IL13Rα1 overexpression (**Supplementary Figure S1**). Together, these data indicated that IL13Rα1 played a tumor suppressor role in PCa.

### IL13Rα1 interacts with and suppresses HK2 expression

**Figure 2:**
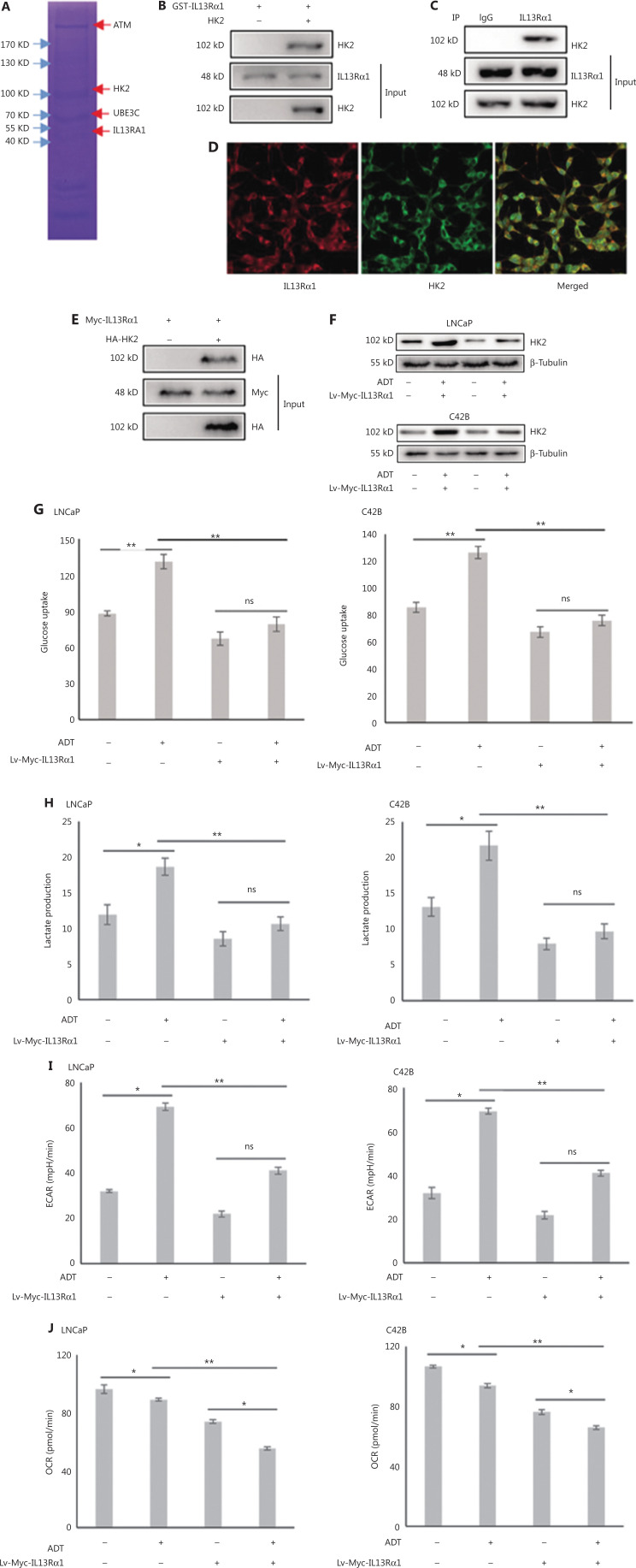
IL13Rα1 inhibits HK2 expression and glycolytic activity. (A) Whole lysates immunoprecipitated with GST-tagged IL13Rα1 were separated by SDS-PAGE for mass spectral analysis. (B) Whole lysates immunoprecipitated with GST-tagged IL13Rα1 were subjected to immunoblotting with anti-HK2. (C) Total protein from LNCaP were co-immunoprecipitated with anti-IL13Rα1 and immunoblotted using anti-HK2. (D) Immunoflurorescence analysis of IL13Rα1 (red) and HK2 (green) in LNCaP. (E) Co-IP from lysates of HEK293T cells co-transfected with plasmids containing Myc-tagged wild type IL13Rα1 and hemaglutinin-tagged HK2. (F) Immunoblot analyses of HK2 in Lv-Myc-IL13Rα1-tranfected LNCaP and C42B with a challenge of ADT for 24 h. (G and H) Glucose concentrations (G) and L-lactate level (H) were monitored in the incubation media of LNCaP and C42B transfected with Lv-Myc-IL13Rα1 with the challenge of ADT for 24 h. (I and J) Extracellular acidification rate and cellular oxygen consumption rates in LNCaP and C42B were measured by the Seahorse XF24 analyzer. Data are representative of 2 independent experiments (B, C, E, and F) or from 3 independent experiments (G–J) and expressed as the mean ± SD. *P < 0.05; **P < 0.01; ns, not significant (two-tail Student’s *t*-test).

To gain mechanistic insight into cell apoptosis induced by IL13Rα1, GST pulldown assays and MS were used to profile the interactions of IL13Rα1 in LNCaP cells. MS analysis showed that IL13Rα1 was co-purified with HK2 (**Figure 2A**), which was confirmed by co-IP analysis showing that GST-tagged IL13Rα1 bound to HK2 in LNCaP cells (**Figure 2B**). Furthermore, we also found HK2 among the endogenous proteins immunoprecipitated by the IL13Rα1 antibody (**Figure 2C**). The IF analysis showed that IL13Rα1 and HK2 co-localized in the cytoplasm of LNCaP cells (**Figure 2D**). To confirm their interaction, Myc-IL13Rα1 and HA-HK2 were co-transfected into HEK293T cells for co-IP analyses, which showed that IL13Rα1 interacted with HK2 (**Figure 2E**). We next analyzed the effects of ADT on HK2 expression and glycolytic activity. As expected, the HK2 protein (**Figure 2F**), glucose uptake (**Figure 2G**), and lactate production (**Figure 2H**) were increased (all, *P* < 0.01) in response to ADT. However, overexpressing IL13Rα1 attenuated the increase in HK2 protein (**Figure 2F**) and glycolytic activity (**Figure 2G, H**) resulting from ADT. To characterize the changes in glycolytic pathways, we measured the ECAR (**Figure 2I**) and OCR (**Figure 2J**) using an extracellular flux analyzer (XF24, Seahorse Bioscience). LNCaP and C42B cells overexpressing IL13Rα1 were characterized by a reduction in ECAR (**Figure 2I**) but an increase in OCRn (**Figure 2J**). Based on these results, we hypothesize that IL13Rα1 might be involved in inhibiting the glycolytic pathway and activating mitochondrial metabolism.

### IL13Rα1 promotes the ubiquitin-mediated degradation of HK2 through UBE3C

**Figure 3:**
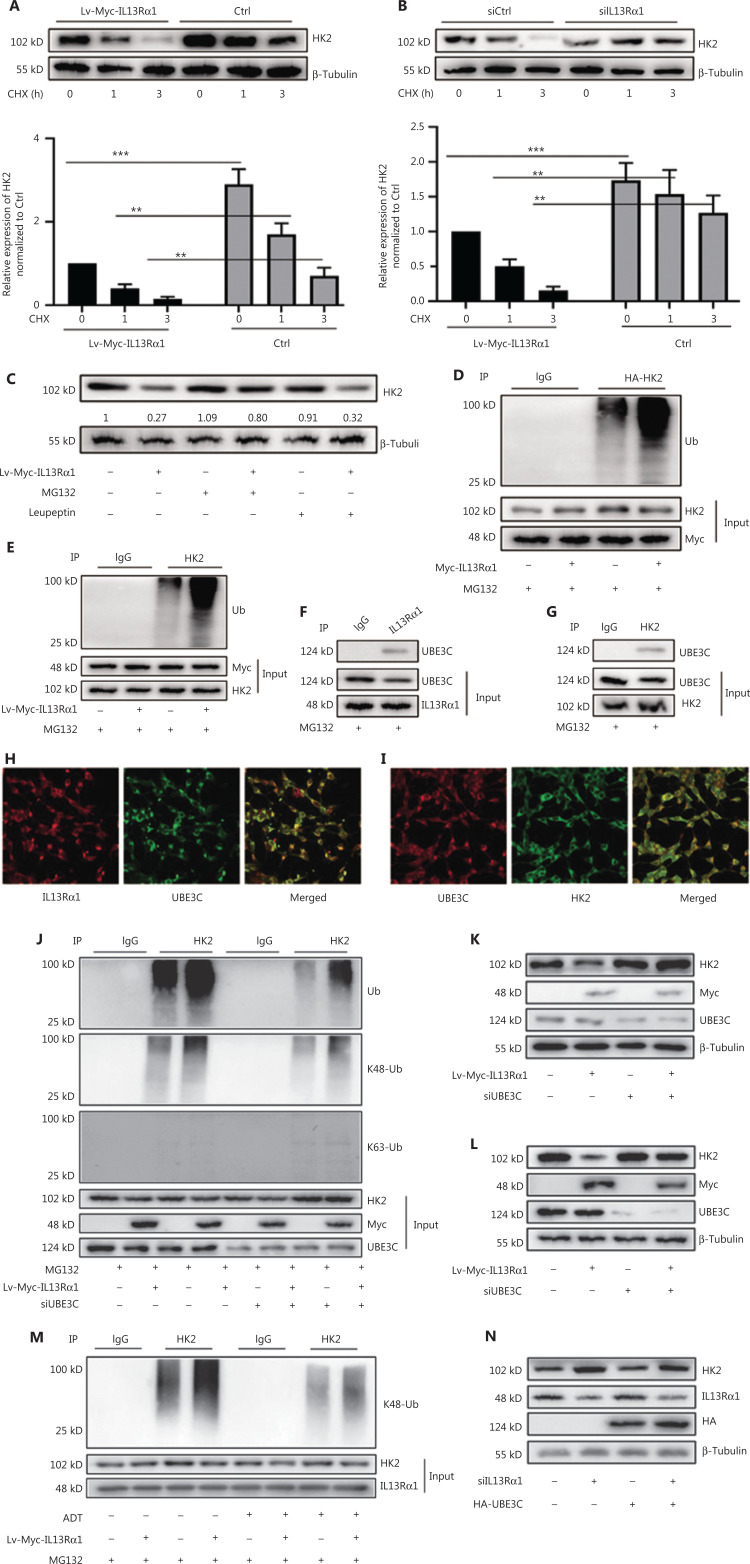
IL13Rα1 promotes the ubiquitin-mediated protein degradation of HK2 through UBE3C. (A) LNCaP harboring Lv-Myc-tagged IL13Rα1 or its control were incubated with cycloheximide (CHX). Western blotting was performed with anti-HK2. (B) siIL13Rα1-, or siCtrl-tranfected LNCaP were treated with CHX. Western blotting was performed with anti-HK2. The protein level of HK2 was quantified by densitometry after normalization to the control. Values are the mean ± SEM. **P < 0.01; ***P < 0.001. (C) LNCaP containing Lv-Myc-tagged IL13Rα1 or its control was treated with MG132 or leupeptin. Whole lysates underwent immunoblotting (IB) with anti-HK2. The band intensity was measured using ImageJ software and presented as the fold change compared with 0 min of CHX (A and B) or without any treatment. (D) Co-immunoprecipitation (Co-IP) from cell lysates of HEK293T cells co-transfected with plasmids encoding Myc-tagged IL13Rα1 and hemaglutinin-tagged HK2 in the presence of MG132. Cell lysates were immunoprecipated with anti-HK2 antibody, followed by IB with anti-Ubiquitin. (E) Co-IP from lysates of LNCaP containing Lv-Myc-IL13Rα1 or Lv-Ctrl with the stimulation of MG132. Cell lysates were immunoprecipitated with anti-HK2 antibody, followed by IB with anti-ubiquitin. (F and G) Co-IP from lysates of LNCaP cells after treatment with MG132. Cell lysates were immunoprecipitated with anti-IL13Rα1 antibody (F) or anti-HK2 antibody (G), followed by western blotting with anti-UBE3C. (H and I) Immunoflurorescence analysis of IL13Rα1 (red)/UBE3C (green), and HK2 (green)/UBE3C (red) in LNCaP cells. (J) Co-IP analysis of HK2 and ubiquitination analyses from lysates of HEK293T cells after the indicated transfections. (K) Western blot analysis of HK2 in LNCaP co-transfected with siUBE3C and plasmids encoding Myc-tagged IL13Rα1. (L) LNCaP harboring Lv-Myc-IL13Rα1 or Lv-Ctrl were transfected with siUBE3C or siCtrl. Western blotting was performed as in (K). (M) LNCaP containing the Lv-Myc-IL13Rα1 or Lv-Ctrl were challenged with androgen deprivation therapy in the presence of MG132. Co-IP was performed to analyze ubiquitination. (N) HEK293T cells transfected with plasmids for HA-UBE3C and siIL13Rα1. Western blotting was performed with anti-HK2, anti-IL13Rα1, anti-HA or anti-β-tubulin. Data are representative of 2 or 3 independent experiments.

Next, we identified the molecular mechanism through which IL13Rα1 inhibited HK2 expression. The CHX chase assay showed that the HK2 protein degraded more rapidly after introducing IL13Rα1 into LNCaP cells (**Figure 3A**). Moreover, there were decreased protein levels recovered after silencing IL13Rα1 (**Figure 3B**). LNCaP cells overexpressing IL13Rα1 were then exposed to MG132 or leupeptin. The downregulation of HK2 as a result of IL13Rα1 was attenuated by the addition of MG132, but not leupeptin (**Figure 3C**), which suggested that the IL13Rα1-induced degradation of HK2 occurred by proteasome-mediated degradation. Further analysis showed that IL13Rα1 overexpression in HEK293T cells led to increased polyubiquitination of HK2 (**Figure 3D**). A similar effect was also observed in Lv-IL13Rα1-transduced LNCaP cells (**Figure 3E**). Notably, MG132 was added to these experiments to protect IL13Rα1 from protein degradation. Thus, the protein degradation of HK2 under androgen-deprived conditions was mediated by the ubiquitin-proteasome pathway.

In our MS data, UBE3C was also identified to be a component of the interactome recruited by IL13Rα1 (**Figure 2A**). The interaction between IL13Rα1 and UBE3C (**Figure 3F**), as well as HK2 and UBE3C (**Figure 3G**), in LNCaP cells, was further confirmed by co-IP (**Figure 3F and 3G**) and IF analyses (**Figure 3H and 3I**). The k48, but not k63 ubiquitin-HK2, was then determined, and the increased ubiquitin levels of HK2 in HEK293T cells induced by IL13Rα1 overexpression were attenuated by silencing UBE3C (**Figure 3J**). Consequently, the inhibitory effect of IL13Rα1 overexpression on the HK2 protein almost disappeared after silencing UBE3C (**Figure 3K**). Similar effects were also observed after silencing UBE3C in LNCaP cells containing Lv-Myc-IL13Rα1 (**Figure 3L and 3M**). More importantly, overexpressing UBE3C in HEK293T cells minimally regulated the HK2 expression that was dysregulated by knocking-down IL13Rα1 (**Figure 3N**). Taken together, these results further supported a role for IL13Rα1 in repressing HK2 through UBE3C-mediated ubiquitination.

### IL13Rα1 recruits ATM to phosphorylate and suppress HK2 expression

**Figure 4:**
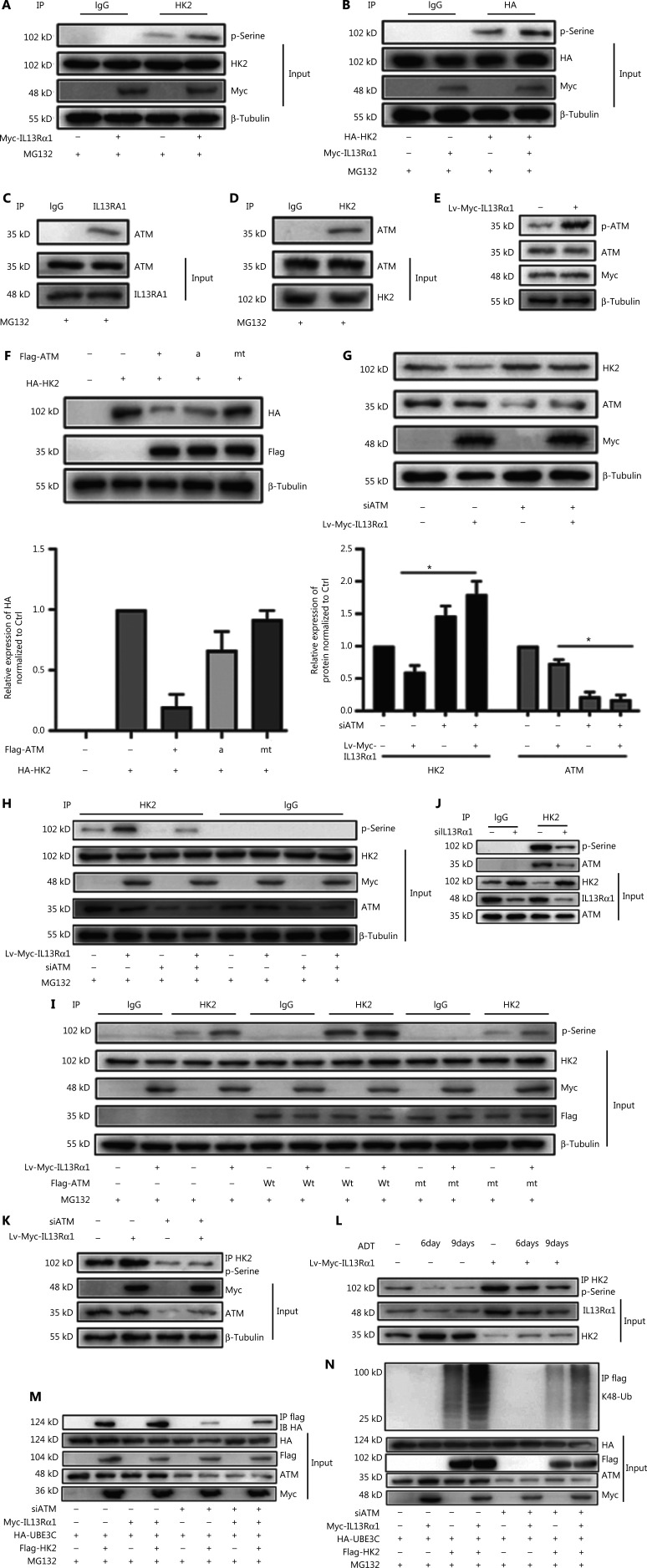
IL13Rα1 recruits ATM to phosphorylate and degrade HK2 protein. (A) Co-immunoprecipitation (Co-IP) from HEK293T cells overexpressing Myc-IL13Rα1 after stimulation with MG132. Whole cells lysates were immunoprecipitated with anti-HK2 and immunoblotted with anti-phosphorylated serine. (B) HEK293T cells were co-transfected with Myc-tagged IL13Rα1 and hemagglutinin (HA)-tagged-HK2. HA-IP protein was subjected to immunoblotting (IB) by anti-phosphorylated serine. (C and D) Whole lysates were immunoprecipitated with anti-IL13Rα1 (C) or anti-HK2 (D) and immunoblotted with anti-ATM, anti-IL13Rα1, or anti-HK2. (E) Immunoblot analyses of phosphorylated ATM in LNCaP that were transfected with Lv-Myc-IL13Rα1 or its control. (F) HEK293T cells were transfected with FLAG-tagged ATM (wild type), active ATM (a) or inactive ATM (mutant), and total protein was subjected to IB using anti-HK2. (G) Western blot analysis of HK2, ATM, Myc, and β-tubulin in Lv-Myc-IL13Rα1 overexpressing LNCaP transfected with siATM. The protein levels of HK2 and ATM were quantified by densitometry after normalization to the control. Values are the mean ± SEM. *P < 0.01. (H) Lv-Myc-IL13Rα1 or Lv-Ctrl transduced LNCaP were transfected with siATM- or siCtrl. Whole cell lysates were immunoprecipitated with anti-HK2 and IB with anti-phosphorylated serine. (I) Co-IP from lysates of HEK293T co-transfected with plasmids Myc-IL13Rα1 along with ATM (wild type), inactive ATM (mutant), or VCtrl. Whole lysates were immunoprecipitated with anti-HK2 and IB with anti-phosphorylated Serine (p-Serine. (J) Co-IP from lysates of siIL13Rα1- or siCtrl-transfected LNCaP. Whole lysates were immunoprecipitated with anti-HK2 and IB with anti-phosphorylated Serine and anti-ATM. (K) Co-IP from lysates of LNCaP containing the Lv-Myc-IL13Rα1 or Lv-Control in the presence of siATM. Whole lysates were immunoprecipitated with anti-HK2 and immunoblotted with anti-phosphorylated p-Serine. (L) Co-IP from lysates of LNCaP containing the Lv-Myc-IL13Rα1 or Lv-Ctrl in response to ADT. Whole cell lysates were immunoprecipitated with anti-HK2 and immunoblotted with anti-p-serine. Data are representative of 2 or 3 independent experiments. (M, N) HEK293T containing plasmids encoding Myc-tagged IL13Rα1, HA-tagged UBE3C, and FLAG-tagged HK2 were further transfected with siATM in the presence of MG132. Whole cell lysates were immunoprecipitated with anti-FLAG followed by IB with anti-HA (M) or anti-K48 ubiquitin (N). Data are representative of 2 or 3 independent experiments.

Protein phosphorylation serves as a molecular signal for ubiquitinating target proteins by using the E3 enzyme complex^19^, and our immunoprecipitation analysis showed that IL13Rα1 induced the serine phosphorylation of both endogenous HK2 in LNCaP (**Figure 4A**) and HA-tagged HK2 (**Figure 4B**) in HEK293T cells. It was reported that ATM regulated cellular responses to DNA damage by phosphorylating multiple substrates, such as p53^20^. Further analysis showed that ATM may account for the phosphorylation of HK2 in response to IL13Rα1 overexpression, based on the following observations. First, ATM was identified as a potential cofactor in the interactome of IL13Rα1 (**Figure 3A**). The interaction between IL13Rα1 and ATM (**Figure 4C**), as well as HK2 and ATM (**Figure 4D**), was further confirmed by co-IP (**Figure 4C and 4D**. Second, the phosphorylation of ATM increased after overexpressing IL13Rα1 in LNCaP cells (**Figure 4E**). Because ATM could phosphorylate and destabilize its targeted protein^21^, we determined whether ATM was responsible for the inhibition of HK2 caused by IL13Rα1 overexpression. We co-transfected the overexpression vectors of wt ATM, and the active ATM or its inactive form with HA-tagged HK2 in HEK293T cells. **Figure 4F** shows a decrease in the HA-tagged HK2 protein in HEK293T cells overexpressing wt ATM or active ATM, but not its inactive form. Conversely, silencing ATM (**Figure 4G**) was sufficient to restore HK2 protein deregulation by overexpressing IL13Rα1 in LNCaP cells. We next determined whether ATM played a role in the phosphorylation of HK2. To this end, siRNA targeting ATM or its negative control was transfected into LNCaP and LNCap-IL13Rα1 cells. Consistently, silencing ATM attenuated the increased levels of phosphorylated HK2 induced by IL13Rα1 overexpression (**Figure 4H**). Furthermore, mt ATM had no effect on the phosphorylation of HK2 (**Figure 4I**). Together, these results suggested that the IL13Rα1 overexpression-induced phosphorylation of HK2 was mediated by ATM.

To further support the possibility that IL13Rα1 interacted with and recruited ATM for HK2 phosphorylation, a co-IP experiment was first performed in siIL13Rα1- or siCtrl-transfected LNCaP cells. Silencing IL13Rα1 resulted in a significant decrease in the interaction between ATM and HK2 (**Figure 4J**) and the serine phosphorylation of the HK2 protein (**Figure 4J**). Furthermore, treatment with siATM or its inhibitor attenuated the increase in the phosphorylated HK2 levels in LNCaP cells induced by IL13Rα1 overexpression (**Figure 4K**). Finally, we analyzed the effects of ADT for different periods (6 and 9 days) on HK2 in Lv-IL13Rα1-transduced LNCaP cells. The levels of phosphorylated HK2 were decreased, which consequently led to an increased level of the HK2 protein in androgen-deprived PCa cells. However, overexpressing IL13Rα1 in PCa cells induced more phosphorylation of HK2 with a resultant decreased level of the HK2 protein (**Figure 4L**). These results showed that IL13Rα1 may be responsible for the ATM-mediated phosphorylation and subsequent degradation of HK2. We then determined whether activation of ATM modulated the physical interaction between UBE3C and HK2. We transfected HEK-293T cells with plasmids encoding FLAG-tagged HK2 and HA-tagged UBE3C and then treated the cells with siATM. Inhibition of ATM blocked the physical interaction between HA-tagged UBE3C and FLAG-tagged HK2 (**Figure 4M**), as well as subsequent ubiquitination (**Figure 4N**). Hence, the association of UBE3C with HK2 required a critical phosphorylation-acceptor site to mediate the ubiquitination and degradation of HK2. These results suggested that ATM phosphorylated HK2 and regulated its stability.

### IL13Rα1 Y402 is essential for its effect on apoptosis

**Figure 5:**
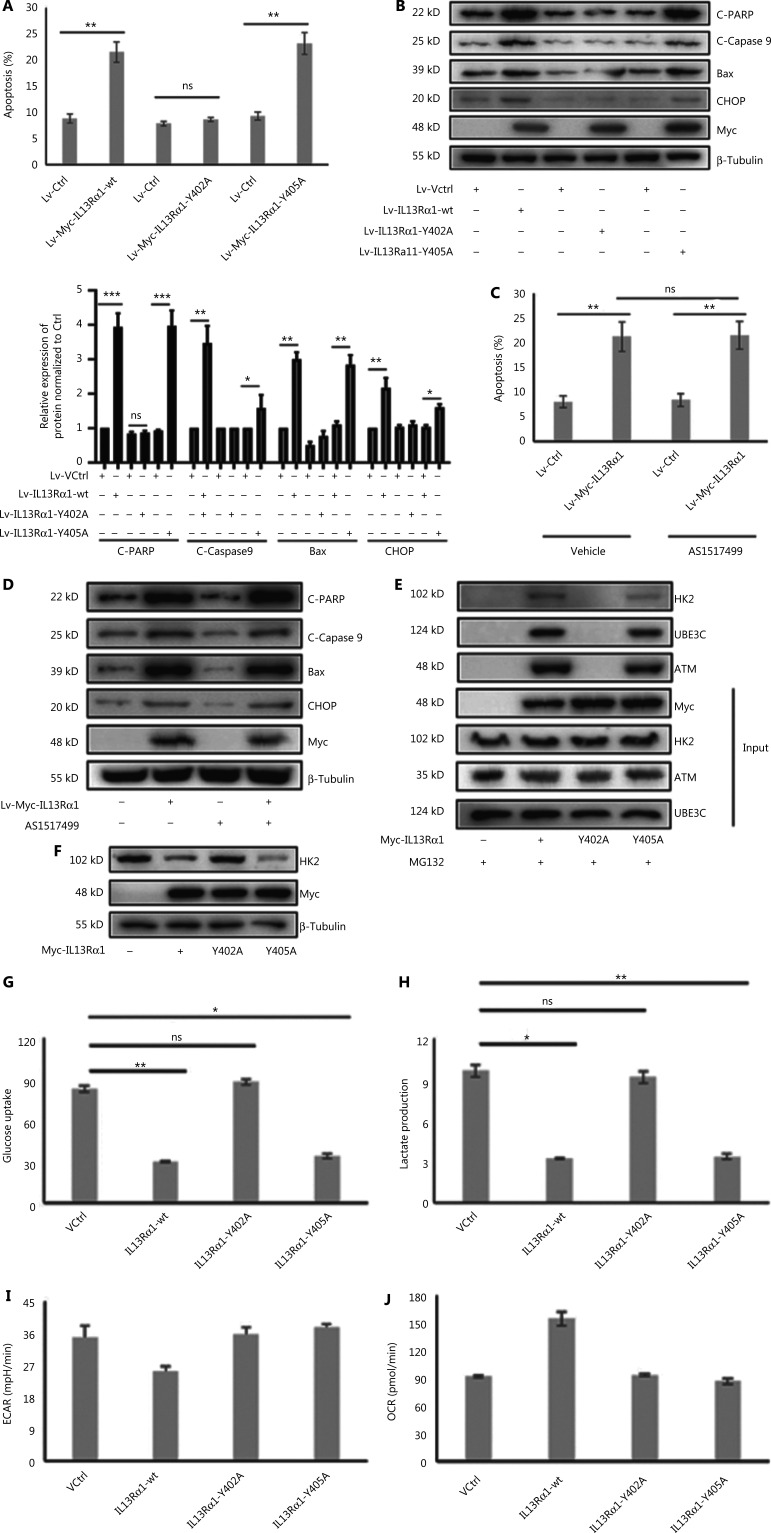
Y402 is essential for the activity for IL13Rα1. (A) The apoptosis of LNCaP containing the Lv-Myc-IL13Rα1 (wt), Lv-Myc-IL13Rα1 (Y402A), Lv-Myc-IL13Rα1 (Y405A), or Lv-Ctrl. (B) Immunoblot (IB) analyses of Bax, cleaved-caspase 9, cleaved-PARP, and CHOP in LNCaP containing the Lv-Myc-IL13Rα1 (wild type), Lv-Myc-IL13Rα1 (Y402A), Lv-Myc-IL13Rα1 (Y405A), or Lv-Ctrl. Quantification of C-PARP, C-Caspase9, Bax, and CHOP levels in b; the control was set at one. *P < 0.05; **P < 0.01; ***P < 0.001. (C) The apoptosis of LNCaP containing the Lv-Myc-IL13Rα1 or Lv-Control was detected with a concomitant treatment with STAT6 inhibitor, AS1517499, for 24 h. (D) Immunoblotting (IB) analyses of Bax, cleaved-caspase 9, cleaved-PARP, and CHOP in LNCaP with treatment with AS1517499. (E) Co-immunoprecipitation (co-IP) from lysates of HEK293T transfected with wild type Myc-IL13Rα1, mutant IL13Rα1 (Y402A) or mutant IL13Rα1 (Y405A). Cell lysates were subjected to IP using anti-Myc, followed by western blotting with anti-HK2. (F) IB analyses of HK2 in HEK293T transfected with Myc-IL13Rα1 in the wild type or mutant type (Y402A and Y405A). (G, H) Glucose concentrations (G) and lactate levels (H) were determined in the incubation media of HEK293T cells transfected with Myc-tagged IL13Rα1 with the wild type or mutant type (Y402A and Y405A). (I, J) The extracellular acidification rate (I) and oxygen consumption rate (J) were measured in HEK293T cells transfected with IL13Rα1 in the wild type or mutant type. *P < 0.05; **P < 0.01; ns, not significant (two-tail Student’s *t*-test).

Previous studies showed that a truncated IL13Rα1 lacking its intracellular domain was unable to mediate IL13-induced signals or responses^10^. The cytoplasmic domain of human IL-13Rα1 contains 2 tyrosine residues, Y402 and Y405, which might serve as docking sites for additional signaling intermediates^10^. We constructed 2 mutant IL13Rα1 proteins (Y402A or Y405A). Importantly, overexpression of mutant IL13Rα1 (Y402A) by lentivirus transduction was unable to induce the apoptosis of LNCaP cells compared with overexpression of the IL13Rα1-wt or mutant IL13Rα1 (Y405A) in the absence of IL13 (**Figure 5A and 5B**). IL13Rα1 was reported to activate STAT6 after binding to IL13. However, inactivating STAT6 with its inhibitor, AS1517499, hardly affected the cell apoptosis caused by IL13Rα1 overexpression (**Figure 5C and 5D**). These results suggested that Y402 was important for the activity of IL13Rα1 and indicated that the presence of distinct IL13Rα1 involved interactome complexes.

Because Y402 was essential for IL13Rα1 activity, we hypothesized that it may function as the docking site for the HK2/ATM/UBE3C complex. As expected, mt Y402A no longer interacted with HK2, ATM, or UBE3C (**Figure 5E**). Functionally, we observed that transfection of the mutant IL13Rα1 (Y402A) into HEK293T cells led to a marked impairment of its suppressive effects on HK2 expression (**Figure 5F**), glucose uptake (**Figure 5G**), and lactate production (**Figure 5H**) compared with the IL13Rα1-wt or mt IL13Rα1 (Y405A). In addition, IL13Rα1 (Y402A) failed to reduce the ECAR (**Figure 5I**) and increased the OCR (**Figure 5J**).

### IL13Rα1/HK2 causes synergistic cytotoxicity and tumor amelioration

**Figure 6:**
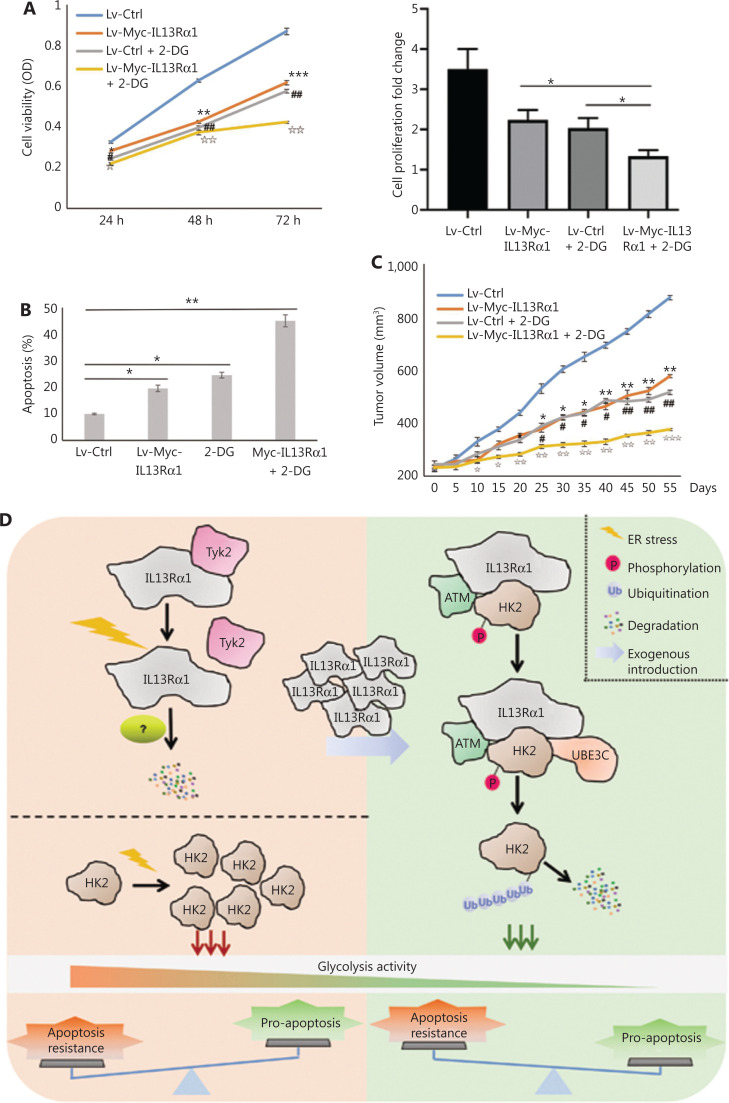
IL13Rα1 in combination with HK2 inhibition synergistically delayed prostate cancer progression under castrated conditions. (A) Cell viability was measured using the MTS assay in C42B cells with the introduction of Lv-Myc-IL13Rα1 or Lv-Ctrl in combination with 2-deoxy-Dglucose (2-DG, 20 mM). *P < 0.05; **P < 0.01; ***P < 0.001: Lv-Myc-IL13Rα1 *vs.* Lv-Ctrl; ^##^*P* < 0.05: Lv-Ctrl+2-DG *vs.* Lv-Ctrl; ^II^*P* < 0.01: Lv-Myc-IL13Rα1+2-DG *vs.* Lv-Ctrl (two-tail Student’s *t*-test). The bar graphs were presented as a fold ratio relative to the control. (B) The apoptosis of C42B cells containing the Lv-Myc-IL13Rα1 or Lv-Ctrl was assessed by Annexin V staining with 2-DG (20 mM) treatment. *P < 0.05; **P < 0.01. (C) Male Balb/c athymic nude mice with or without castration, and 5 × 10^6^ cells/100 mL of C42B transduced with Lv-Myc-IL13Rα1 or Lv-Ctrl were injected subcutaneously. The mice were then treated with 2-DG (800 mg/kg) and tumor volumes were measured on the indicated days. The number of animals in each group was 10. *P < 0.05, **P < 0.01: Lv-Myc-IL13Rα1 *vs.* Lv-Ctrl; ^#^*P* < 0.05, ^##^*P* < 0.05: Lv-Ctrl+2-DG *vs.* Lv-Ctrl; ^I^*P* < 0.05, ^II^*P* < 0.01, ^III^*P* < 0.001: Lv-Myc-IL13Rα1+2-DG *vs.* Lv-Ctrl (two-tail Student’s *t*-test). Data are from 2 or 3 independent experiments. (D) Proposed mechanism of IL13Rα1 driven apoptosis of PCa. IL13Rα1 protein dissociated with Tyk2 and degraded more rapidly under androgen deprived conditions. This led to an enhancement of HK2 and consequent glycolysis, which may contribute to apoptosis resistance against androgen deprivation. After introduction of IL13Rα1 in PCa cells, more HK2s were recruited and destined for ubiquitin-proteasomemediated protein degradation, which finally resulted in a decrease of glycolysis and subsequent apoptosis of PCa cells. Furthermore, IL13Rα1 acted as a scaffold for HK2, ATM, and UBE3C. ATM phosphorylated IL13Rα1 and facilitated UBE3C-mediated ubiquitination. IL13Rα1 represented a novel factor for specific glycolysis inhibition and inhibited apoptosis resistance against androgen deprivation.

A previous study confirmed the therapeutic potential of IL13R cytotoxicity and HK2 inhibition in PCa. In the present study, we further confirmed that C42B cells treated with the combination of IL13Rα1 overexpression and 2-DG exhibited synergistic cytotoxicity, as shown by a decrease (*P* < 0.01) in cell proliferation (**Figure 6A**) but an increase in cell apoptosis (*P* < 0.01) (**Figure 6B**), compared with the above-described treatments alone. To further confirm the important translational implications of the present study, we determined whether IL13Rα1 overexpression potentiated the effect of 2-DG treatment using *in vivo* models under castration conditions. C42B cells were transfected with IL13Rα1, followed by 2-DG treatment. **Figure 6C** shows that overexpressing IL13Rα1 in combination with 2-DG treatment resulted in a further reduction in tumor burden when compared with IL13Rα1 overexpression or 2-DG alone. Together, these results suggested that combined IL13Rα1 overexpression and HK2 inhibition synergistically delayed CRPC growth both *in vitro* and *in vivo*.

## Discussion

Molecular changes, including restored androgen receptor (AR) signaling, AR bypass signaling, and complete AR independence, lead to CRPC^1^. However, inhibition of apoptosis can also contribute to the resistance of PCa to androgen deprivation. IL13Rα1 has been reported to regulate cellular responses to TGF-β-mediated fibrosis and oxidative damage^22,23^. In the present study, gain- and loss-of-function experiments showed that IL13Rα1-mediated activity served as a novel molecular program to promote apoptosis in response to androgen deprivation. The decreased stability of the IL13Rα1 protein, possibly resulting from its dissociation from Tyk2 in response to ADT, may have contributed to the transition of PCa cells from androgen dependence to independence. It is thus anticipated that targeting IL13Rα1 may provide a novel treatment strategy for PCa, especially CRPC.

The Warburg effect occurs in PCa cells as an adaptation that alters the existing cell metabolism and supports cell growth^24^. In response to androgen deprivation, cell survival is sustained by metabolic adaptation to aerobic glycolysis^25^. Therefore, inhibiting glycolytic metabolism may promote a shift from anti-survival to pro-apoptosis programs under androgen-deprived conditions, which also has therapeutic implications. HK2 catalyzes the first committed step of glucose metabolism and has been recognized as an oncogenic kinase^14^. Several lines of evidence suggest that HK2 is an important component for fine tuning the balance between pro-apoptosis and anti-apoptosis effects^26^. HK2 could protect against mitochondrial-regulated apoptosis through interaction with voltage-dependent anion selective channels^26^. Moreover, HK2 localized in mitochondria can confer apoptotic resistance against Bax/Bak^15^ and inhibit Ca^2+^- and ROS-induced mitochondrial permeability transition pore opening and cell death^27–29^. Recently, Bustamante et al. and our study showed that an increase in HK2 was closely related to the dysregulated apoptosis of fibroblasts from rheumatoid arthritis patients, and targeted inhibition of HK2 was an attractive treatment for arthritis^26^. In the present study, we identified a novel pro-apoptosis pathway involving IL13Rα1 in PCa, which inactivated the HK2-mediated glycolytic process under androgen-deprived conditions, indicating the likelihood of IL13Rα1-mediated signaling as a treatment target for PCa.

HK2 serves as an important substrate of posttranslational modification, as shown by the proliferation of leukocytes being decreased when HK2 is ubiquitinated and degraded^30^. Furthermore, the Lys63-linked ubiquitination of HK2 catalyzed by the E3 ligase, TRAF6, is critical for its selective autophagy degradation^31^. Ubiquitin-protein ligase E3C (UBE3C) promotes both cancer growth and metastasis by activating the Wnt/β-catenin pathway^32^ or increasing the epithelial-mesenchymal transition^33^. Moreover, UBE3C promotes glioma progression by ubiquitinating and degrading Annexin A7^34^. In the present study, we observed that UBE3C targeted HK2 for K48-linked polyubiquitination, reduced HK2 stability, and consequently induced PCa cell apoptosis under androgen-deprived conditions. Additionally, it has been reported that ATM deficiency promoted the progression of CRPC by enhancing the Warburg effect^16^. However, the exact mechanism underlying the inhibitory role of ATM in glycolysis remains unknown. Here, HK2 was identified as a novel phosphorylated substrate for ATM, and ATM-mediated phosphorylation promoted ubiquitin-mediated protein degradation of HK2. The negative regulation of HK2 by ATM was also shown by ursolic acid promoting the apoptosis of tumor cells by inhibiting HK2-mediated glycolysis, but activating ATM^35^. Collectively, our study suggested that ATM and UBE3C coordinately and specifically targeted HK2 for protein degradation using IL13Rα1 as a bridge, thereby promoting the apoptosis of PCa cells.

## Conclusions

The data obtained from *in vitro* studies on androgen-deprived PCa cells together with the data from *in vivo* experiments in which IL13Rα1-overexpressing PCa cells were injected into castrated mice enabled us to conclude that IL13Rα1 inhibited glycolysis to disrupt the apoptosis resistance in response to androgen deprivation. Regarding a possible treatment, we showed that ectopic expression of IL13Rα1 plus HK2 inhibition additively inhibited tumor growth under castration resistance conditions. Together, our findings revealed a novel function of IL13Rα1 in suppressing glycolysis in PCa and also provided an alternative option to overcome apoptosis resistance to androgen deprivation, as well as a novel treatment strategy for PCa (**Figure 6D**).
