# Supplementary material for: IL13Rα1 prevents a castration resistant phenotype of prostate cancer by targeting hexokinase 2 for ubiquitin-mediated degradation
Source: Cancer Biol Med. 2021 Oct 18;19(7):1008–28. doi: 10.20892/j.issn.2095-3941.2020.0583 (PMC9334759; doi:10.20892/j.issn.2095-3941.2020.0583)
Supplement: Supplementary file 1 [file cbm-19-1008-s001.pdf]

Supplementary materials

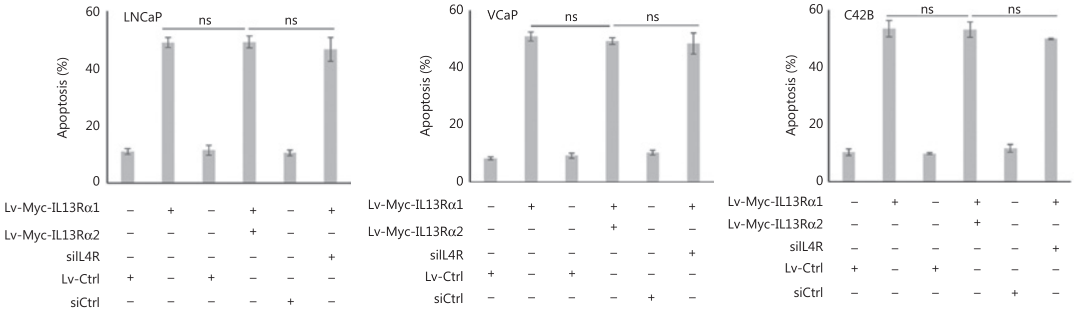

Figure S1 The effects of IL4Rα and IL13Rα2 on IL13Rα1-induced apoptosis. The apoptosis of LNCaP containing the lentiviral-Myc-tagged IL13Rα1 (Lv-Myc-IL13Rα1) or its control (Lv-Ctrl) was assessed by Annexin V staining with IL13Rα2 overexpression or IL4Rα knockdown. ns, not significant (two-tail Student's *t*-test).

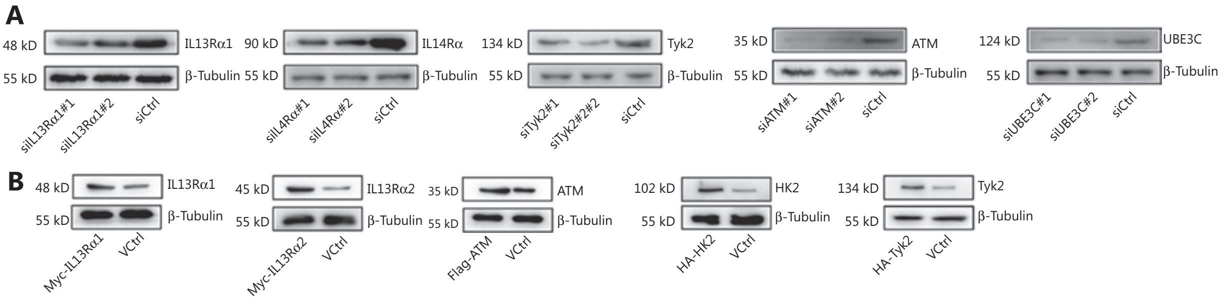

Figure S2 (A) Expression of IL13Rα1, IL13Rα2, HK2, ATM, and Tyk2 was detected in HEK293T cells after transfection with indicated plasmids. (B) The silencing efficiency of various siRNAs.

Table S1 Details of the siRNAs used in this study

siRNA	Sequence
IL13Rα1#1	GGAAACTCGTCGTTCAATA
IL13Rα1#2	GGAGCCAGCTCAAATTGTA
IL4Rα#1	CCAGTCCTCTGAACTCAGA
IL4Rα#2	GCAGATCCTTCACATGAGT
Tyk2#1	GAACTGGCATGGCATGAAT
Tyk2#2	GCACAAGGACCAACGTGTA
ATM#1	GAAAGAGAATGGATTAGAA
ATM#2	GCAACATACTACTCAAAGA
UBE3C#1	CGCAGACCGTTTTCCCTTA
UBE3C#2	TGAGAATGCTTGAAGTATT

Table S2 Primary antibodies

Antibody	Concentration	CAS Number	Company
HA	1:1000	sc7392	Santa-Cruz
Myc	1:1000	2276, A14	Santa Cruz
Flag	1:3000	F1804	Sigm
Ubiquitin 1	1:1000	sc8017	Cell Signaling Technology
K48-linkage polyubiquitin	1:1000	8081	Abcam
K63-linkage polyubiquitin	1:1000	5621	Cell Signaling Technology
IL13Rα1	1:1000	ab79277	Abcam
IL13Rα1	1:1000	sc101382	Santa-Cru
IL4Rα	1:1000	sc-28361	Santa Cruz
IL13Rα2	1:1000	11059	Proteintech
Hexokinase II	1:1000	ab104836	Abcam
Hexokinase II	1:1000	22029	Proteintec
β-tubulin	1:1000	1006	Proteintech
Bax	1:1000	50599	Proteintech
CHOP	1:1000	15204	Proteintech
Caspase 9	1:1000	66169	Proteintec
PARP1	1:1000	13371	Proteintech
Tyk2	1:1000	sc-5271	Santa Cru
UBE3C	1:1000	ab177511	Abcam
UBE3C	1:1000	ab68225	Abcam
Anti-phosphoserine	1:1000	ab9332	Abcam
ATM	1:1000	ab199726	Abcam
ATM	1:1000	sc53173	Santa-Cruz
p-ATM	1:1000	ab81292	Abcam
